# Effects of Er:YAG Laser on Mineral Content of Sound Dentin in Primary Teeth

**DOI:** 10.1155/2014/578342

**Published:** 2014-08-14

**Authors:** Cigdem Guler, Meral Arslan Malkoc, Veli Alper Gorgen, Erhan Dilber, Mehmet Bulbul

**Affiliations:** ^1^Department of Pediatric Dentistry, Faculty of Dentistry, Ordu University, 52100 Ordu, Turkey; ^2^Department of Prosthodontics, Faculty of Dentistry, Inonu University, 44280 Malatya, Turkey; ^3^Department of Pediatric Dentistry, Faculty of Dentistry, Inonu University, 44280 Malatya, Turkey; ^4^Department of Prosthodontics, Faculty of Dentistry, Sifa University, 35100 İzmir, Turkey; ^5^Department of Prosthodontics, Faculty of Dentistry, Eskişehir Osmangazi University, 26040 Eskişehir, Turkey

## Abstract

The aim of the present study was to evaluate the mineral content of sound dentin in primary teeth prepared using an Er:YAG laser at two different power settings. Thirty-six primary second molars were used in this study. Three dentin slabs were obtained from each tooth, and the slabs were randomly divided into three groups: Group A, control; Group B, Er:YAG laser at 3.5 W, 175 mJ, and 20 Hz, short pulse mode; and Group C, Er:YAG laser at 4 W, 200 mJ, and 20 Hz, medium-short pulse mode. One dentin slab per group was used to evaluate the dentinal morphology and surface roughness values using SEM and profilometer, respectively. Mineral content in the dentin slabs were calculated by inductively coupled plasma-atomic emission spectrometry (ICP-AES). The data were analyzed by one-way analysis of variance and Tukey's HSD tests. No significant differences in Ca, K, Mg, Na, and P levels or Ca/P ratio were found among the groups (*P* > 0.05). SEM micrographs showed that surface irregularities increased with a higher power setting. The surface roughness after laser treatment in Group B and Group C was found to be similar, unlike Group A.

## 1. Introduction

Dental caries can lead to pain, infection, pulp necrosis, and tooth loss; as such, it is still considered the most prevalent oral disease during childhood and adolescence [1–3].

Cavity preparation traditionally can be performed based on mechanical and biological principles using nonrotatory and rotatory instruments. However, mechanical techniques can cause vibration, pressure, noise, and pain. Pain may be reduced by local anesthesia, but needles can also cause fear; thus, these techniques usually cause anxiety and stress in pediatric patients [4–6]. Dental pain and fear may be decreased using lasers in dentistry. It has been reported that using an erbium: yttrium aluminium garnet (Er:YAG) laser for cavity preparation produces minimal vibration and noise, minimal or no need for local analgesia, reduction of stress, and minimal removal of sound tooth structure, as well as providing a better surface for adhesive restorative materials [6–8]. Therefore, the use of lasers in pediatric dentistry has increased recently [9–11].

Laser technology can be used in soft tissue surgery, caries prevention, caries diagnosis, cavity preparation, and endodontic treatment for children. Cavity preparation procedures have used different laser systems such as Er:YAG and erbium, chromium: yttrium scandium gallium garnet (Er,Cr:YSGG). However, changes in the dentinal morphology of primary teeth resulting from laser exposure have been reported in the literature. Zhang et al. [12] reported cracks and microfissures in the dentin surface of primary teeth prepared using a high-powered Er:YAG laser. In a different study, Zhang et al. [13] evaluated the dentinal morphology of primary teeth prepared using the Er:YAG laser with different power parameters, and they reported that there was no smear layer and the dentinal tubules were clear. In addition, they found dentin melting and cracks associated with high-powered Er:YAG lasers [13].

There have been recent reports in the literature regarding the mineral content of dental hard tissue prepared by different laser treatments [14–20]. In addition, Ari and Erdemir [19] reported that the adhesion of dental restorative materials to hard tissue was affected by changes in the mineral content of dentin. Therefore, change in the mineral content of dentin is important to restorative practice, bonding mechanism, and microleakage. In recent studies on the mineral contents of dental hard tissue prepared by different laser treatments, permanent teeth have been used [14–20]. Because the mineral contents of enamel and dentin in primary teeth are different from those of permanent teeth, the effect of laser on the mineral content of primary teeth may be different from that of permanent teeth as well.

There are limited studies related to mineral content of dental hard tissue in primary teeth [21–23]. there are some reports related to the concentration of trace element in primary teeth of children with healty, different conditions and/or syndromes [24–28]. In addition, However, the effect of Er:YAG laser on the mineral content of sound dentin in primary teeth has not been studied yet.

The aim of the present study was to evaluate the mineral content of sound dentin in primary teeth prepared using an Er:YAG laser at two different power settings. The null hypothesis tested was that there would be differences in the mineral content—levels of calcium (Ca), potassium (K), magnesium (Mg), sodium (Na), and phosphorus (P) and Ca/P ratio—of sound dentin in primary teeth prepared using an Er:YAG laser at two different power settings.

## 2. Material and Methods

### 2.1. Sample Preparation

All sample preparation was performed by the same operator (V.A.G.) to prevent interoperator variation. The study samples were comprised of 36 human lower primary second molars that were free of dental caries or restoration and extracted for orthodontic reasons. The study was approved by the Faculty of Medicine, Noninvasive Clinical Research Ethic Committee, Ordu University (2013-34). After cleaning, the teeth were mounted 1 mm above of cementoenamel junction, vertically in quadrangular molds with an autopolymerizing acrylic resin (Meliodent; Bayer Dental, Newbury, UK). Tooth enamel was removed with a diamond high speed cylinder bur under water cooling manually because of dentin layer being thin. The cut surface of each tooth to confirm the absence of enamel was digitally observed under light microscope, magnification X20 (Olympus SZ4045 TRPT, Osaka, Japan). Then, the occlusal thirds of the crowns were cut using a slow-speed diamond saw (Isomet; Buehler, Lake Bluff, IL) under water cooling. Three 0.6-mm thick, dentin slabs were obtained from each tooth for the same mineral concentrations [17] and randomly divided into three groups (*n* = 36 per group):

 Group A: control group, no treatment.
 Group B: dentin irradiated with an Er:YAG laser at 3.5 W, 175 mJ, and 20 Hz, short pulse mode.
 Group C: dentin irradiated with an Er:YAG laser at 4 W, 200 mJ, and 20 Hz, medium-short pulse mode.

### 2.2. Laser Treatment

All laser applications were performed by the same operator (M.B.) to prevent interoperator variation. The laser probe was held in the same position so that it did not move across the tooth surface.

Two standard laser device modes (Fidelis Plus 3; Fotona, Ljubljana, Slovenia) were used for the laser treatment. The first, 3.5 W, 175 mJ, and 20 Hz, short pulse mode, was used for Group B. In this group, the laser was applied with a sapphire probe (1 mm in diameter); the contact handpiece (R14) was placed perpendicular to the surface at a distance of 1 mm, and the entire dentin area was scanned at a speed of 1 mm/sec with water and air cooling. For this application, the device power was set at 3.5 W with short pulse mode (300 *μ*s), and the repetition rate was 20 Hz with 175 mJ pulse energy.

The second, 4 W, 200 mJ, and 20 Hz, medium-short pulse mode, was used for Group C. In this group, the laser was applied with 4 W power (200 mJ pulse energy with 20 Hz repetition rate) and with a medium-short pulse (100 *μ*s).

The average time of laser irradiation in each group was 30 seconds per specimen.

### 2.3. Inductively Coupled Plasma-Atomic Emission Spectrometry (ICP-AES) Technique

ICP-AES experimental procedures were conducted according to Malkoc et al. [14, 15] and Secilmis et al. [16].

All dentin slabs (*n* = 34 per group) were stored at 70°C in cabinet desiccators (Ventisell, Italy) until they reached a constant weight. Then each specimen was weighed on an electronic balance (AX200; Shimadzu Corporation, Kyoto, Japan) and the weight was recorded [14]. Nitric acid (10 mL) and hydrochloric acid (3 mL) were added onto the specimens and they were digested in a microwave reaction system (Mars 5; CEM, Matthews, NC) at 180°C and 180 psi [16].

After calibration of the ICP-AES instrument (Vista AX, Varian, Mulgrave, Australia), 2 mL of solution was taken. The solutions are carried in a nebulizer with the help of a peristaltic pump. The specimen is turned into an aerosol which is carried by an argon spray. The aerosol is heated by conduction and radiation and reaches approximately 10,000°C. Light is transferred to a detector, and every element is evaluated according to its wavelength. Five measurements were performed on each element for each solution, and the means of the measurements were calculated in milligrams per liter (parts per million) [15, 16]. The levels of Ca, K, Mg, Na, and P in each specimen were determined, and the mineral contents were then calculated as percentage by weight.

### 2.4. Scanning Electron Microscopy (SEM) Examinations

All dentin slabs (*n* = 1 per group) were prepared for SEM (LEO EVO 40 VP; Leo Electron Microscopy Ltd., Cambridge, UK). After surface treatment, the specimens were coated (BAL-TEC SCD 050 sputter coater; BAL-TEC AG, Balzers, Liechtenstein) with gold/palladium, and micrographs were obtained.

### 2.5. Surface Roughness Examinations

All dentin slabs (*n* = 1 per group) were prepared for surface roughness examinations. The mean surface roughness values (Ra) of study samples were evaluated using a profilometer (Surf Test-402 surface roughness tester; Mitutoyo Corp., Tokyo, Japan) in all groups. To measure the roughness value in micrometers, a diamond stylus (tip radius, 5 lm) was moved across the surface under a constant load of 0.75 mN with a speed of 0.5 mm/sec and a range of 350 lm. The instrument was calibrated using a standard precision reference specimen. Three traces were recorded for each specimen at three different locations in different positions (parallel, perpendicular, and oblique) giving nine tracings per sample. The average of these nine mean surface roughness measurements was used as the score for each sample. The scores were entered into a spreadsheet (Excel; Microsoft, Seattle, WA) for calculation of descriptive statistics.

### 2.6. Statistical Analysis

Statistical analysis of the data was performed using SPSS 16.0 for Windows (SPSS, Chicago, IL). The differences in mineral content between the groups were analyzed with one-way analysis of variance (ANOVA), and the comparison of means was performed with the Tukey HSD multiple comparisons test. Statistical differences were determined at a 95% confidence level (*P* = 0.05).

## 3. Results

### 3.1. ICP-AES Evaluation

The mean percentage weights of the five elements (Ca, K, Mg, Na, and P) in the dentin slabs are shown in Table 1. One-way ANOVA showed that there were no significant differences between Ca, K, Mg, Na, and P levels or Ca/P ratio (*P* > 0.05) among the three groups.

### 3.2. SEM Evaluation

**Figure 1:**
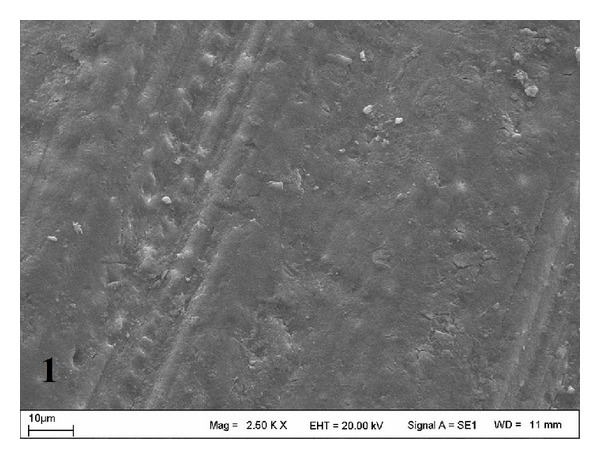
SEM micrograph of dentin surface in control group (original magnification ×2.50 k).

**Figure 2:**
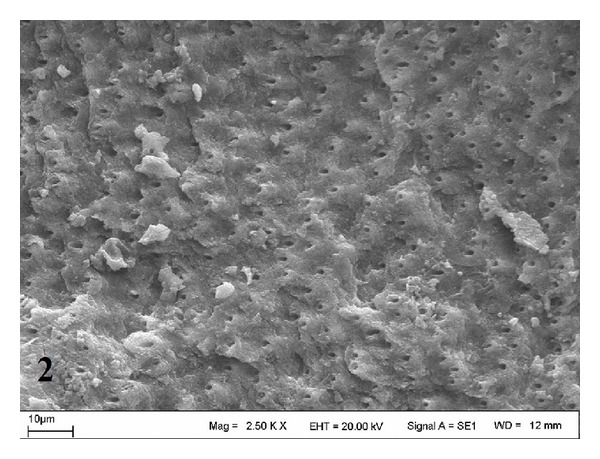
SEM micrograph of dentin surface treated with 3.5 W laser in Group B (original magnification ×2.50 k).

**Figure 3:**
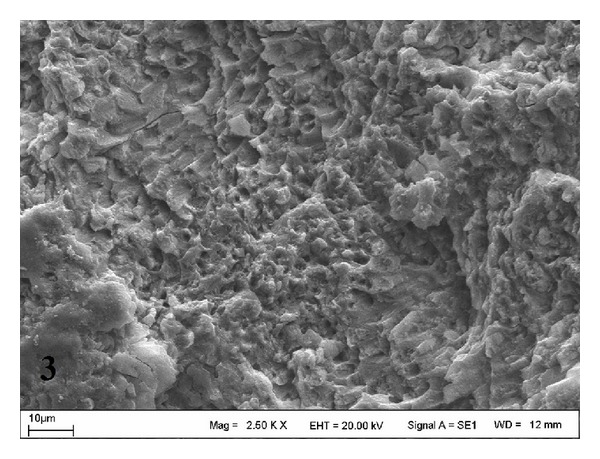
SEM micrograph of dentin surface treated with 4 W laser in Group C (original magnification ×2.50 k).

SEM views of the control (Group A) are shown in Figure 1. Er:YAG laser-treated dentin surfaces (Groups B and C) are shown in Figures 2 and 3. The surface treated at Group C was rougher than those of Groups A and B. A rough, crystalline irradiated dentin surface could be observed in both slabs treated with the laser. This appearance was more pronounced in the Group C (Figure 3).

### 3.3. Surface Roughness Evaluation

The mean surface roughness values of all groups are shown in Table 2. The lower mean surface roughness value was found in Group A. The higher mean surface roughness value was found in Group C. However, similar mean surface roughness values were found in laser treatment groups (Groups B and C).

## 4. Discussion

In this study, the compositional changes (Ca, K, Mg, Na, and P) in the sound dentin in primary teeth prepared by an Er:YAG laser at two different power settings were evaluated and the Ca/P ratios of the groups were compared using ICP-AES. The mean percentage weights of Ca, Mg, Na, and P and the Ca/P ratio of the groups were not affected by laser irradiation. Therefore, the null hypothesis was rejected.

To evaluate the mineral content of dental hard tissue, different methods can be used, such as energy dispersive spectrometer (EDS), wavelength dispersive X-ray fluorescence spectrometry (WDXRF), atomic absorption spectrophotometer (AAS), and FT-Raman spectroscopy [18, 29–31]. However, the ICP-AES technique was preferred for this study due to the following advantages: (1) this technique is one the most attractive methods for the measurement of trace elements; (2) trace elements can be measured at the micrograms per liter level; and (3) multiple elements can be measured at the same time [19, 32].

Dentinal morphology is important when a tooth needs to be treated with adhesive restorative materials. The cavity preparation with the Er:YAG laser could be an alternative for fearful children in pediatric dentistry. However, use of the Er:YAG laser in both primary and permanent teeth might cause changes in dentinal morphology. Dentin consists of organic and inorganic components; the major inorganic components of dental hard tissue are Ca and P present in hydroxyapatite crystals. It has been reported that the Ca/P ratio of dentin depends on factors such as hydroxyapatite crystal type, the availability of Ca, the anatomic location, and the technique used [19, 33, 34]. In addition, it has been reported that altering the Ca/P ratio of dentin can change the morphology of dentin and affect the adhesion of restorative materials to dental hard tissues [19, 35]. According to results of this study, the Ca/P ratios in the dentin of primary teeth were not affected by Er:YAG laser irradiation at two different power settings. However, SEM photographs indicated that the surface irregularities increased when the power setting was increased. Secilmis et al. [16] reported that surface irregularities in the dentin of permanent teeth increased when a high-powered laser was used. These results were in accordance with our findings. However, they found that Ca, Mg, Na, and P levels and Ca/P ratio in the dentin of permanent teeth were affected by Er,Cr:YSGG laser treatment. These results were not in accordance with our findings and might be related to the use of permanent teeth and Er,Cr:YSGG laser. Also, in the present study, surface roughness was increased after laser treatment. The higher mean surface roughness value was found in Group C: 4 W, 200 mJ, and 20 Hz, medium-short pulse mode. However, similar mean surface roughness values were found in laser treatment groups (Groups B and C). The increase of surface roughness value may be related to energy density of the Er:YAG laser. However, our surface roughness results should be supported by further studies using more dentin slabs.

A few studies have been conducted on the effect of Er:YAG laser on the dentinal micromorphology of primary teeth. In these laser studies, different power settings of Er:YAG laser in primary teeth were used. Kornblit et al. [36] evaluated the enamel and dentin morphology of primary teeth using an Er:YAG laser with different power parameters. They observed no carbonization or cracks, and the SEM micrographs were similar to those of permanent teeth. Flury et al. [37] compared the dentinal morphology of primary teeth using an Er:YAG laser and a diamond bur, and they reported open dentin tubules for all the laser-treated groups. These results were in accordance with our findings; we observed open dentin tubules for both laser groups when compared to the control group. However, our SEM results should be supported by further studies using more dentin slabs.

The mineral content of sound dentin in primary teeth prepared using “3.5 W, 175 mJ, and 20 Hz, short pulse mode,” and “4 W, 200 mJ, and 20 Hz, medium-short pulse mode,” of Er:YAG laser was evaluated in this study. Laser parameters used in the study were predetermined by the manufacturer's instructions. The same parameters of Er:YAG laser in primary teeth have not been studied yet. However, similar power setting of Er:YAG laser in primary teeth was used in a few studies [12, 13, 37, 38]. Zhang et al. [13] reported that laser power of less than 4 W at Er:YAG laser should be used for cavity preparation in primary teeth. In addition, they found that the use of the laser at 10 Hz/200 mJ and 10 Hz/300 mJ for cavity preparation in primary teeth is safe and effective. Monghini et al. [39] reported that Er:YAG laser irradiation of dentin in primary teeth adversely affected bond strength. In addition, they found that the increase of laser energy resulted in increasingly cratered surfaces. These results were in accordance with our findings.

Most of the studies on the effect of dental lasers on the mineral content of hard tooth tissues have been carried out on permanent teeth [14–20]. However, there are some differences between primary and permanent tooth dentin, such as thickness, number of dentin tubules, degree of mineralization, and inorganic component. The concentration of Ca and P in peritubular and intertubular dentin is lower in primary teeth than in permanent teeth. In this study, it was found that the mineral content of sound dentin in primary teeth was not affected by laser irradiation. It may be that the two laser power settings of Groups B and C did not lead to different mineral content, but they did vary in surface irregularities. The surface irregularity in Group C was higher than that in Group B. Mineral content of sound dentin in primary teeth may be change with different power settings of Er:YAG laser. Therefore, correlations among Er:YAG laser power settings, mineral content, surface irregularities, and roughness during laser preparation of dentin in primary teeth should be investigated in further studies.

From a clinical viewpoint, there are limitations concerning the correlation between in vitro and in vivo tests and also clinical usage. There are some limitations of this in vitro study. First, only two laser power settings of Er:YAG laser were used in this study. Mineral content of sound dentin in primary teeth may change using different power settings of Er:YAG laser. Second, only Er:YAG laser was used in this study. Mineral content of sound dentin in primary teeth may change using different laser type. Third, surface irregularities and roughness were evaluated in this study using only one dentin slab per group. The surface irregularities and roughness results may change using more dentin slabs.

## 5. Conclusion

Laser irradiation makes structural and chemical changes on the dental hard tissues. These changes alter the level of solubility and permeability of dentin. Consequently, the bond strength of adhesive systems on dentine surfaces may be affected in clinical practice. However, laser treatment did not affect the mean percentage weights of Ca, K, Mg, Na, and P or the Ca/P ratio in any group of primary teeth in present study. Therefore, tested power settings of the Er:YAG laser is safe for cavity preparation in primary teeth.

## Figures and Tables

**Table 1:** Mean percentage weights of the five elements (mean ± standard deviation) and Ca/P ratio according to groups (*n* = 34 per group).

Groups	Ca	K	Mg	Na	P	Ca/P
Group A	21.554 ± 1.605	0.054 ± 0.013	0.587 ± 0.165	0.646 ± 0.094	9.302 ± 0.734	2.328 ± 0.220
Group B	22.385 ± 1.442	0.060 ± 0.016	0.647 ± 0.092	0.614 ± 0.060	9.260 ± 0.386	2.415 ± 0.06
Group C	23.075 ± 1.538	0.059 ± 0.014	0.621 ± 0.118	0.685 ± 0.052	9.545 ± 0.417	2.415 ± 0.07
*P* values	0.103	0.651	0.579	0.100	0.450	0.284

**Table 2:** Mean surface roughness values according to groups (nine tracings per sample).

Groups	Roughness value (means ± standard deviation)
Group A	0.41 ± 0.2
Group B	1.68 ± 0.3
Group C	1.90 ± 0.2
